# Eye-Tracker-Guided Non-Mechanical Excimer Laser Assisted Penetrating Keratoplasty

**DOI:** 10.3390/s130303753

**Published:** 2013-03-18

**Authors:** Edgar Janunts, Frank Schirra, Nora Szentmáry, Berthold Seitz, Achim Langenbucher

**Affiliations:** 1 Institute of Experimental Ophthalmology, Saarland University, Homburg/Saar 66421, Germany; E-Mail: achim.langenbucher@uks.eu; 2 Department of Ophthalmology, Saarland University Medical Center, Homburg/Saar 66421, Germany; E-Mails: frank.schirra@uks.eu (F.S.); nszentmary@hotmail.com (N.S.); berthold.seitz@uks.eu (B.S.)

**Keywords:** eye-tracker, trephination mask, computerized penetrating keratoplasty, non mechanical corneal trephination, excimer laser

## Abstract

*Purpose*: The purpose of the study was to implement a new eye tracking mask which could be used to guide the laser beam in automated non-mechanical excimer laser assisted penetrating keratoplasty. *Materials and methods*: A new trephination mask design with an elevated surface geometry has been proposed with a step formation between conical and flat interfaces. Two recipient masks of 7.5/8.0 mm have been manufactured and tested. The masks have outer diameter of 12.5 mm, step formation at 10.5 mm, and slope of conical surfaces 15°. Its functionality has been tested in different lateral positions and tilts on a planar surface, and pig eye experiments. After successful validation on porcine eyes, new masks have been produced and tested on two patients. *Results*: The build-in eye tracking software of the MEL 70 was always able to capture the masks. It has been shown that the unwanted pigmentation/pattern induced by the laser pulses on the mask surface does not influence the eye-tracking efficiency. The masks could be tracked within the 18 × 14 mm lateral displacement and up to 12° tilt. Two patient cases are demonstrated. No complications were observed during the surgery, although it needs some attention for aligning the mask horizontally before trephination. Stability of eye tracking masks is emphasized by inducing on purpose movements of the patient head. *Conclusion*: Eye-tracking-guided penetrating keratoplasty was successfully applied in clinical practice, which enables robust tracking criteria within an extended range. It facilitates the automated trephination procedure of excimer laser-assisted penetrating keratoplasty.

## 1. Introduction

Over the last 20 years, excimer laser penetrating keratoplasty (PKP) has been performed by using elliptical or round trephination masks for donor and patient with eight orientation teeth/notches, where the laser beam is manually guided along the edge of trephination masks [1–5]. The excimer laser-based approach has been introduced as an alternative to the mechanical trephination method, and its superiority and specificities such as reduced keratometric astigmatism, higher regularity of topography, improved visual acuity and other features have been reported extensively in the literature [6,7]. Based on the same approach, we have recently introduced a new computerized technique for excimer laser-assisted PKP using the MEL 70 laser (Carl-Zeiss Meditec, Jena, Germany) [8]. Round metal masks aperture for both donor and patient corneas are still used to ensure vertical donor-host-junction and better matching of the graft in the host [9]. In the new approach, the cornea is perforated along the edge of the metal mask in an automatic way.

**Figure 1.**
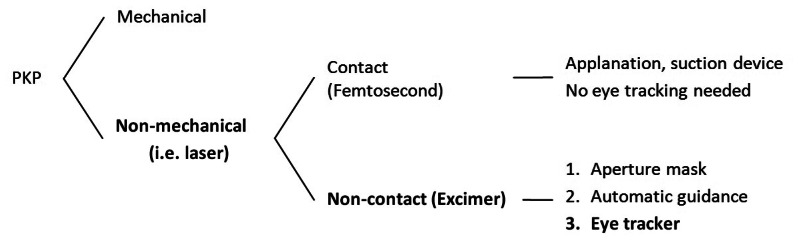
Schematic overview of current approaches in penetrating keratoplasty (PKP) with respect to eye tracking necessity and availability.

The logic consequence of the automatization process for the laser trephination procedure was the integration of an eye tracker (Figure 1), since fast misalignments of the patient eye as well as periodic movements (heart beat or breathing) cannot be compensated fully by the surgeon [10,11]. To our knowledge, PKP assisted by an eye tracker has never been performed. Therefore, no publications have been found in the literature on eye-tracker-guided corneal transplantation. Moreover, in most types of lamellar keratoplasty the cornea is applanated during the incision (irrespective of whether mechanical or laser trephines are used). Therefore, there is no need for eye tracking (Figure 1). However, corneal applanation should be avoided in penetrating keratoplasty and the clinical interest demands non-contact means for corneal trephination. In this study, we introduced an active eye tracking feature to the computerized non-mechanical excimer laser-assisted PKP.

The purpose of the study was to implement a new eye tracking mask which could be recognized by the build-in eye-tracker of the MEL70 excimer laser, and could be used to guide the laser beam in automated non-mechanical excimer laser-assisted PKP.

## 2. Materials and Methods

A planar metal ring mask is normally used as a reference object for the eye tracker in MEL70 for refractive surgery. Since in automated laser-assisted PKP a metal mask is also used, it has been decided to leave the camera optics and software intact, and instead, to design a new trephination metal mask which could be detected by the eye tracker software without additional changes in the standard settings of the MEL70 software.

Although eye tracking is not needed for donor trephining, since it is performed on a stationary stage and there are no movements expected, the conventional PKP donor mask (as described in [9]) is suitable for eye tracking without modifications, but since the conventional recipient mask geometry was not suitable for the eye tracking of the MEL 70 excimer laser, a recipient mask has been newly designed to enable eye-tracking for non-mechanical excimer laser-assisted PKP.

The MEL 70 is factory equipped with an active eye tracking unit for refractive surgery which is controlled by image processing software and a built-in PC. It uses a lightweight metal ring mask positioned on the limbus as a reference object. The build-in eye tracker is based on a monochrome 8 bit video CCD camera (the intensity is divided between 0 and 255 gray values) with a resolution of 752 × 582 pixels. It searches for a circular pattern with contrast gradient from light to dark radially towards the image center. It defines a so called “hot zone” where the pupil center needs to be found, unless the laser pulses will be stopped.

**Figure 2.**
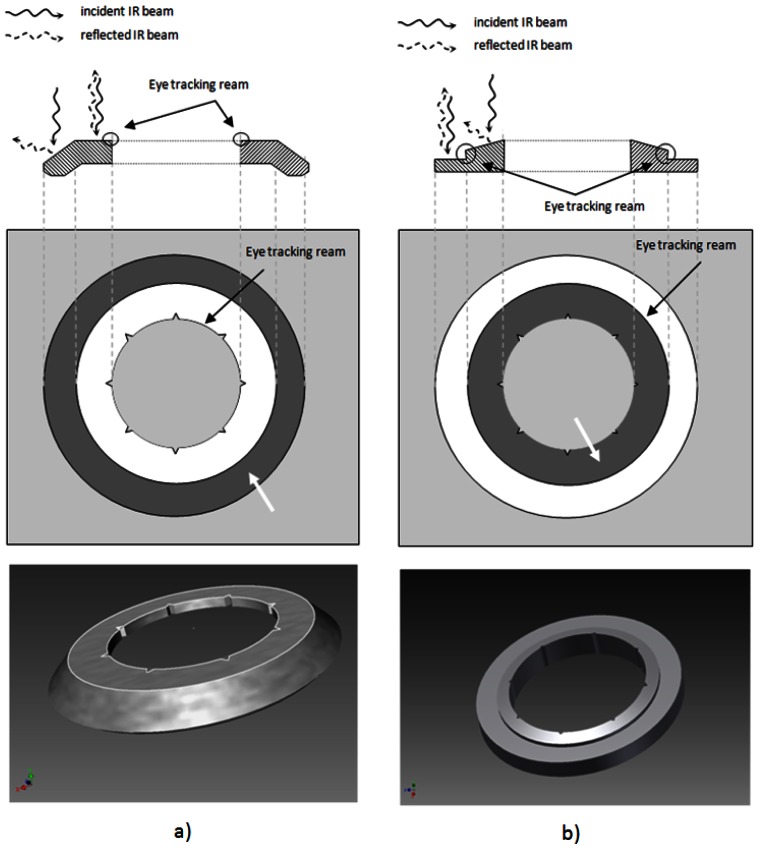
Two different eye-tracking mask designs: (**a**) tracking is reached at the inner-most aperture, (**b**) tracking is achieved in the middle ring. The tracking outlines where the IR contrast changes from bright to dark are highlighted. The white arrows show the circular regions where the mask appears dark in the IR reflected image due to the surface tilt.

Based on the above mentioned concept, two different eye tracking mask designs with different surface geometries have been tested during the development process (Figure 2). Since the eye tracker operates on detection of a reflected IR image and searching for a circular pattern, an elevated surface design has been proposed with a step formation between conical and flat interfaces. In a reflection image the flat surface appears as bright ring (because of higher reflection), and consequently the conical surface as dark ring, since almost no light gets detected (Figure 2, arrows).

**Figure 3.**
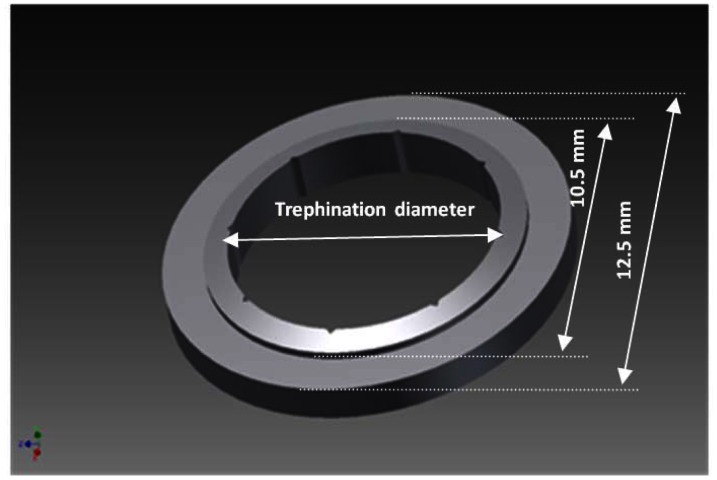
Technical specifications of the eye-tracking-guided trephination mask.

The masks have been manufactured by the VisioTec company (Adelsdorf, Germany) using stainless steel according to our CAD drawing and specifications (Figure 3). Two most often used sizes of recipient masks have been manufactured and tested for clinical applicability: 7.5 mm and 8.0 mm, which correspond to 7.6 mm and 8.1 mm donor masks, respectively. Additionally, 6.0 mm masks have also been manufactured and tested for robustness and proof of principle, although this has not been considered for further clinical use. The masks have an outer diameter of 12.5 mm, the step formation was at 10.5 mm in diameter, and the inner diameter is the trephination diameter. The step formation is an interface of flat and conical surfaces with a slope angle of 15°. The thicknesses of the masks differ at the periphery (due to the conical structure of the inner ring) depending on their effective diameters. The orientation notches were replicated exactly as described in the literature [9]: eight triangular shaped orientation notches sized (0.30 mm in base and 0.15 mm in height) and corresponding teeth at the donor mask.

The following development and testing steps were accomplished:

1. technical design of eye tracking/trephination masks,
2. experimental validation on the plane surface,
3. pig eye experiments,
4. re-manufacturing the masks including eight orientation notches (for clinical use),
5. clinical tests on two patients.

The masks have been tested on plane surfaces, and then on pig eyes. The masks were only used for experimental setup. Pig eyes were obtained from a slaughter house, and were used no more than 12 hours after enucleation. The globes were fixed on cylindrical holders made of rubber. After successful validation on porcine eyes, new masks have been manufactured, including the well known eight orientation notches, for final clinical evaluation and later introduced into clinical practice.

It was not the scope of this work to test the eye-tracking performance during the dynamic movements of the masks. For that we relied on the built-in eye tracking software capabilities to follow the masks appropriately. Therefore, we restricted ourselves to static test conditions.

**Figure 4.**
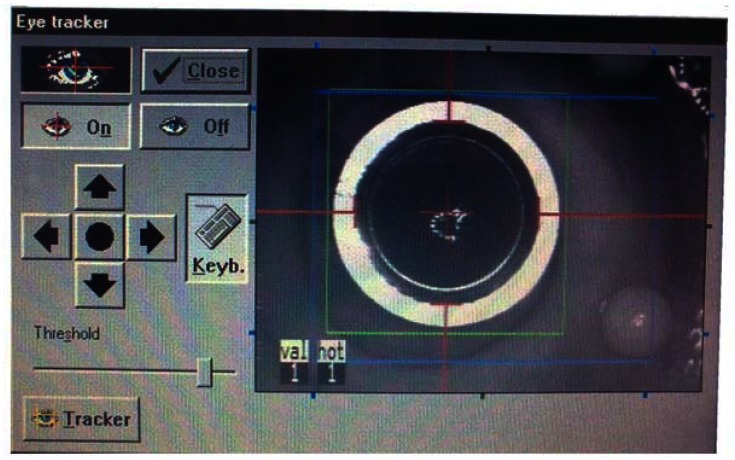
Pop-up diagram in the MEL 70 excimer laser for managing the eye-tracking features. It allows enabling as well as disabling the active eye-tracking by “On” and “Off” buttons, fine alignment of the tracking outlines, and adjusting the threshold value of the IR illumination.

Mask alignment and eye tracking procedure: The mask is positioned on the cornea approximately perpendicular to the optical axis, and after enabling the eye-tracking feature by pressing the “On” button from the corresponding pop-up diagram on the screen (Figure 4), the tracker captures the mask automatically. Any minor mismatch of the detected ring from the real edge of the mask could be corrected via the operating software by the four direction arrow buttons (Figure 4). Additionally, the build-in software allows adjusting contrast and gain of the reflected IR image by changing the threshold value. The threshold has been kept constant for all tests (after adjusting the contrast at the beginning) to check for robustness.

## 3. Results

**Figure 5.**
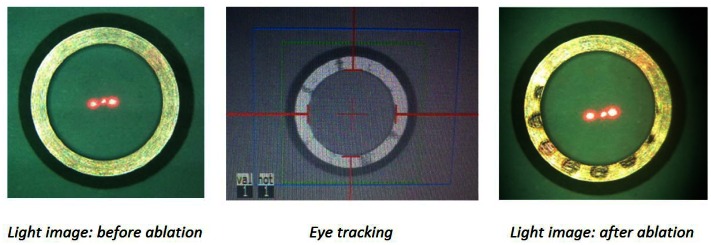
Eye tracking mask with 8 mm inner diameter according to design in Figure 2(a). Laser pulses induce pigmentation on the flat surface of the mask which may interfere with the eye tracking function.

Laser masks with different diameters of 6.0/7.5/8.0 mm are manufactured according to the design data depicted in Figure 2. Both mask designs were recognized by the eye-tracker, but one of them (Figure 2(a)) has been influenced by the pattern induced by the laser ablation (Figure 5). A pigmentation pattern is induced by the laser, which has influenced the tracking efficiency. Therefore, the first mask design has not been evaluated further, although it had been shown to work properly with the eye tracker. In contrast, the second design had more robust performance and was further tested on pig eye globs for the proof of concept.

**Figure 6.**
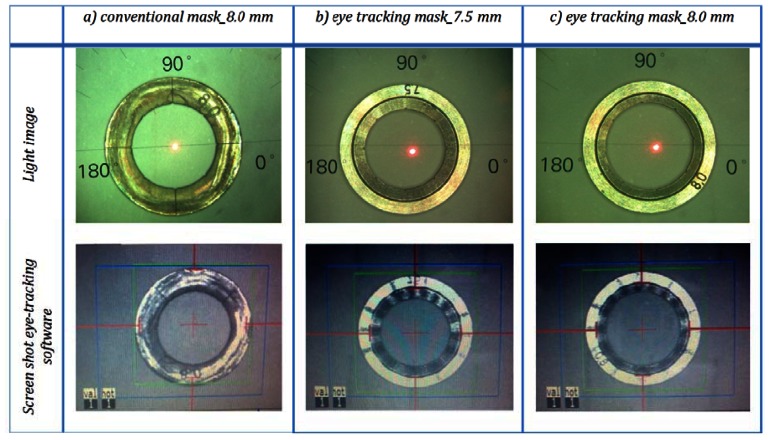
Eye tracking for the conventional *vs.* newly proposed trephination masks. The masks bear eight orientation notches.

As shown in Figure 6, the build-in eye tracking software of the MEL 70 was always able to capture the new proposed trephination masks of all sizes described in Figure 2(b); however the conventional mask is not recognized appropriately (Figure 6(a)). Although the conventional mask fits entirely in the tracking camera field of view (blue rectangular area), the software was unable to capture it correctly. Various illumination conditions create changing outlines for the tracking criteria, and therefore conventional corneal trephination mask in its current form was unsuitable for eye tracking. In the proposed design not only the entire diameter has been reduced (by 0.5 mm), but also a special 3D geometry is engraved on it, which enabled robust criteria of capturing and the mask movements can be observed within an extended range. In Figure 6 the eye tracking of the new trephination masks (including the eight orientation notches) of 7.5/8.0 mm of inner diameter are given. Since the 6.0 mm masks have been manufactured for laboratory tests only, no orientation notches have been engraved.

**Figure 7.**
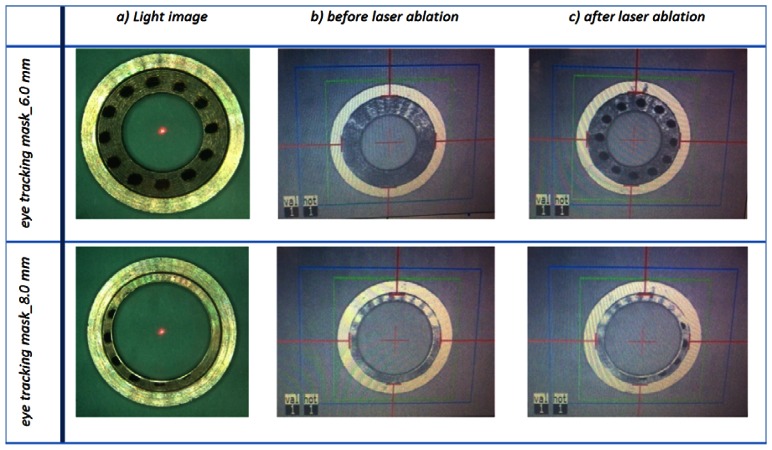
Eye-tracking masks of 6.0 mm and 8.0 mm before and after the first time laser ablation. It is seen that the patterns do not exceed the dark region of the IR reflected image.

All masks have been tested before and after laser ablation as shown in Figure 7, considering the fact that laser pulses cause unwanted pigmentation/patterns on the mask surface, which could potentially interfere with the eye tracking software (as seen in case of conventional mask and the one shown in Figure 5—based on Figure 2(a) design data).

Because of a small beam size in MEL 70 (1.2 mm in diameter), and the minor displacements of the beam during the fine adjustment process, is not expected that the laser beam will meet the bright outer ring of the mask. Figure 7 addresses this issue, where an 8.0 mm ablation profile has been intentionally fired onto the 6.0 mm mask. It demonstrates that even in case of inappropriate large ablation profiles there is enough distance away from the capturing outline (at 10.5 mm diameter).

**Figure 8.**
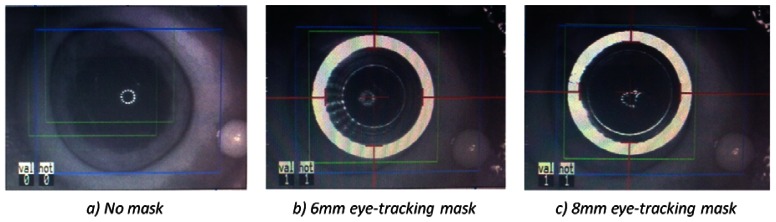
(**a**) depicts the camera view of the porcine eye without any tracking mask. Eye-tracking on the porcine eye using the new trephination masks of 6.0 mm (**b**) and 8.0 mm (**c**).

**Figure 9.**
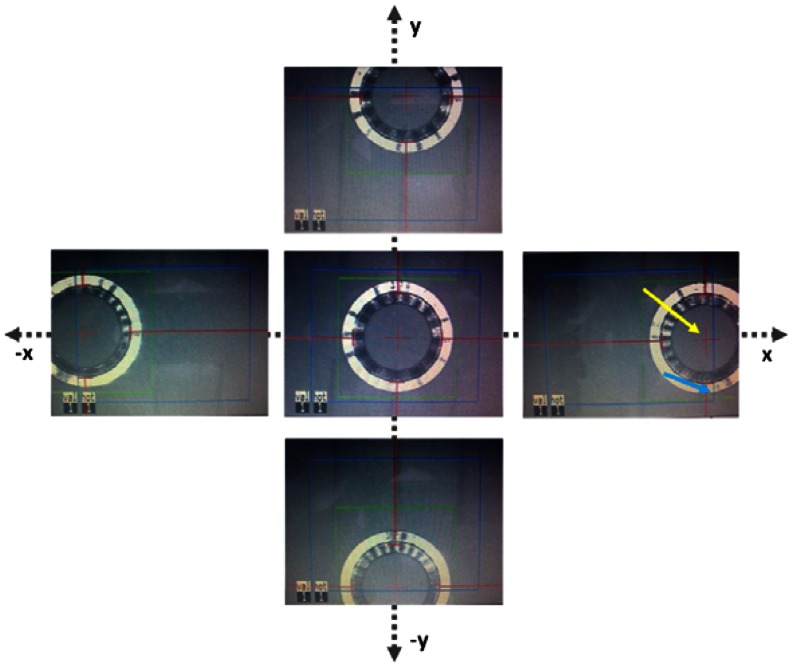
Demonstration of the eye tracker by using the new trephination mask for various lateral displacements on planar surface. The 7.5 mm mask was tracked in horizontal and vertical directions (X-Y). The mask was captured by the eye tracker as far as its center (see yellow arrow) was still in the hot zone (blue rectangle, see blue arrow).

**Figure 10.**
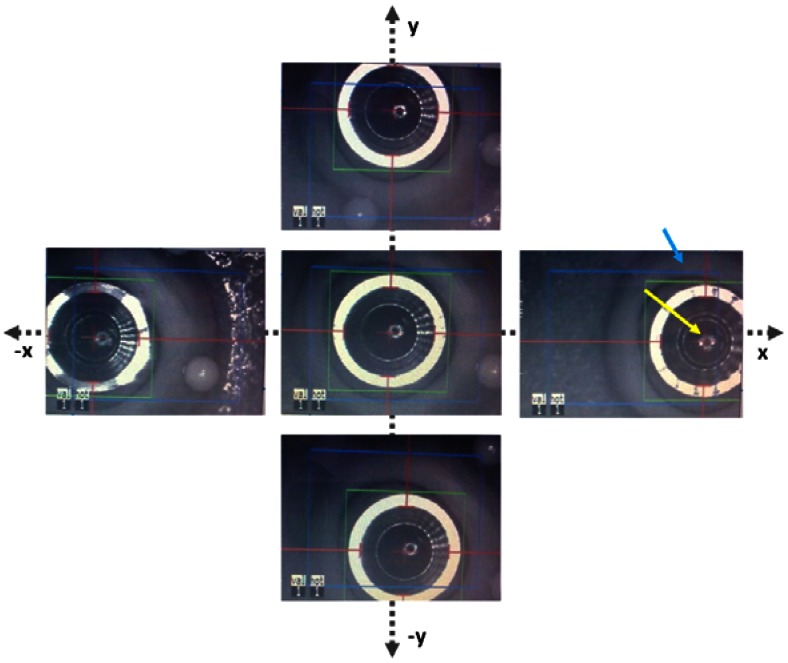
Demonstration of the eye tracker by using the new trephination mask for various lateral displacements placed on the surface of a pig eye globe. The 6.0 mm mask was used here. The mask was recognized by the eye tracker while moving in X-Y directions as far as the mask center (see yellow arrow) was still in the hot zone (blue rectangle, see blue arrow).

The masks have also been tested on porcine eye globes in order to imitate natural background contrast similar to human eye as shown in Figure 8. The masks were easily recognized by the eye tracking software. The red lines/arrows outline the mask position in perpendicular meridians and the green circle the entire mask, respectively. The functionality of the new trephination masks has been tested in different lateral positions and tilts on a planar surface (Figure 9), as well as on the pig eye experiments (Figure 10).

The studies showed that the masks could be tracked within the 18 × 14 mm lateral displacement and up to 12° tilt, since large tilts cause inhomogeneous illumination of the white outer ring. Therefore, it is recommended to align the patient head properly to ensure perpendicular position of the trephination mask (maximum reflection and homogeneous illumination).

**Figure 11.**
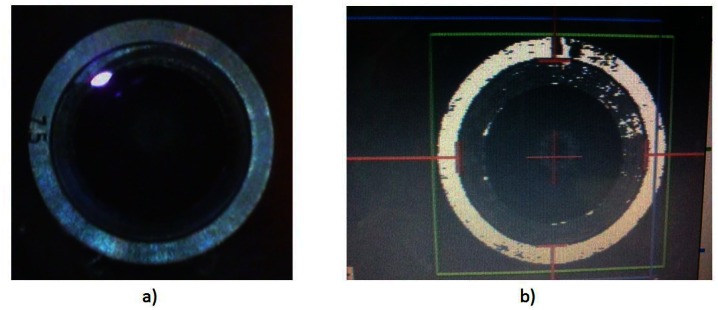
Eye-tracking on a patient eye using 7.5 mm trephination mask. (**a**) microscope image and (**b**) a snapshot image of the eye-tracking software window from the screen.

The eye tracking masks have been tested also on two patients (Figure 11). No complications were observed during the surgery, although some attention is needed for aligning the mask horizontally before trephination. Stability of the eye tracking masks is emphasized by inducing on purpose movements of the patient head in both video streams (Video 1, Video 2, online version of the manuscript), where the laser beam has been following the movements of the mask. Two patients have been treated by different surgeons using the new eye-tracking trephination masks.

## 4. Discussion

There are different implementation strategies for eye tracking [12–15]. The tracking ensures that the reaction time is essentially shorter than typical movements of the eye during surgery, meaning that the next laser shot will be fired faster than essential movements occur [16]. Various systems are commonly used by different companies: in the previous generation of eye trackers only pupil lateral displacement (2D tracking systems) has been addressed. Nowadays, newly developed systems are equipped with sophisticated tracking features, primarily with closed loop feedback. In recent years, the eye registration has also been introduced to clinical practice [17], being more sophisticated but at the same time technically challenging and very time consuming, since the image processing in a shorter time frame is required: sampling rate is more than 10 times the bandwidth. For the registration usually landmarks on the eye which do not change with lighting conditions, such as the limbus, peripherial iris, or reference marks placed by surgeon have been used [13]. Up to now eye tracking systems in ophthalmology register eye movements in the IRIS plane, hence tracking of the corneal surface was required for our purpose. In contrast to the MEL 70 system, which is equipped with a closed loop eye-tracking system, the open loop systems neglect eye movements during the image capture/processing [18].

Up to now, the alignment of the laser beam generally has been done by the surgeon using an aiming beam (pilot laser), which was positioned onto the center of the mask while the excimer beam is in standby mode. For that continuous monitoring (and corrections if needed) of the laser beam path along the mask interface was necessary. After introducing eye tracking trephination masks, excimer laser- assisted PKP becomes even more efficient: After centering the mask according to limbus and approximately perpendicular to the optical axis (laser beam), the tracking system automatically identifies the edges, positions the beam accordingly, and follows the potential movements.

It has been seen that tilt of the mask from the optical axis of the laser may induce inhomogeneous illumination, resulting in some mismatch of the detected ring from the real edge of the mask. Nevertheless, this mismatch could be corrected via the operating software. It is unlikely to expect laterally or rotationally larger displacements than mentioned above during the surgery.

The conventional recipient mask outer diameter of 13 mm has been used [9], which is larger compared to the new eye tracker masks for the MEL 70, limited by the field of view. The larger mask diameter was initially intended to prevent the sclera from being ablated, since the previous generation of lasers were used with a manual beam manipulation. After establishing the automatic approach of corneal trephination, the trephination mask could have already been optimized, since the MEL 70 yields narrower beam size and supports a precise beam manipulation along the trephination edge.

But even reduction of the mask diameter would not be sufficient, since the laser spots create ablation patterns on the mask surface (see Figure 6(a)), which disturb the homogeneous IR image of the mask surface leading to a incorrect assignment of the tracking landmarks. Moreover, these patterns are changing overtime, so a new mask design with robust tracking criteria was required.

As mentioned earlier, the conventional donor mask was compatible to the build-in eye tracking system due to its round geometry, where the metal surface appears dark in the IR image and provides a changing contrast from bright to dark in its outer edge of the mask. The mask geometry was within the dynamic range of tracking.

The trephination mask geometry has been optimized by introducing a 3D geometry instead of a flat surface in order to achieve stable eye tracking even at a smaller diameter of 10.5 mm. Moreover, the overall diameter has been reduced slightly (only 0.5 mm), to protect the sclera from laser ablation.

## 5. Conclusions

This the first time that an eye-tracking-guided penetrating keratoplasty was performed. The use of eye tracking masks represents a useful technical refinement of automated excimer laser-assisted PKP. It even further facilitates the automated trephination procedure. Since the PKP eye tracking using the MEL 70 is performed without modifying the built-in software or any hardware changes, the method could easily be translated into regular clinical routine and may also be implemented easily by other Ophthalmology Departments.
